# The impact of preoperative nutritional status on postoperative outcomes: an insight from Geriatric Nutritional Risk Index in elderly pancreaticoduodenectomy patients

**DOI:** 10.1186/s12893-024-02397-0

**Published:** 2024-04-05

**Authors:** Teng-Yuan Hou, Yu-Hung Lin, Yueh-Wei Liu, Yu-Yin Liu, Wei-Feng Li, Ming-Chun Kuo, Szu-Wei Huang, Cheng-Hsi Yeh, Yu-Cheng Lin, Shih-Min Yin

**Affiliations:** 1 Division of General Surgery, Department of Surgery, College of Medicine, Kaohsiung Chang Gung Memorial Hospital and Chang Gung University, 123 Ta-Pei Road, Niao-Song, Kaohsiung, 833 Taiwan; 2 Division of Hematology Oncology, Department of Internal Medicine, College of Medicine, Kaohsiung Chang Gung Memorial Hospital and Chang Gung University, Kaohsiung, Taiwan; 3 Department of Obstetrics and Gynecology, College of Medicine, Kaohsiung Chang Gung Memorial Hospital and Chang Gung University, Kaohsiung, Taiwan

**Keywords:** Malnutrition, Pancreatoduodenectomy, Geriatric Nutritional Risk Index

## Abstract

**Background:**

Malnutrition is not uncommon among the elderly undergoing pancreatoduodenectomy (PD) and is related to increased complications. Previous studies have shown that the Geriatric Nutritional Risk Index (GNRI) predicts outcomes in various populations. Nevertheless, the research exploring the correlation between GNRI and postoperative outcomes in PD is scarce. This study aimed to investigate the preoperative malnutrition, as measured by GNRI, on outcomes in elderly patients undergoing PD.

**Materials and Methods:**

This retrospective analysis enrolled 144 elderly patients underwent PD for periampullary tumors from November 2016 to December 2021. Patients were stratified based on the GNRI value: high/moderate nutrition risk (GNRI ≤ 92, *N* = 54), low nutrition risk (92 < GNRI ≤ 98, *N* = 35), and no nutrition risk (GNRI > 98, *N* = 55). Perioperative outcomes and postoperative surgical complications were compared between these groups. Univariate and multivariate analyses were performed on major postoperative complications and prolonged postoperative length of stay (PLOS).

**Results:**

Patients in the high/moderate risk group were significantly older, with lower BMI (*P* = 0.012), higher mortality rate (11.1%, *P* = 0.024), longer PLOS (*P* < 0.001), and higher incidence of over grade IIIB complications (37.0%, *P* = 0.001), Univariate and multivariate analyses showed the high/moderate risk GNRI group (OR 3.61, *P* = 0.032), increased age (OR 1.11, *P* = 0.014) and operative time over 8 h (OR 3.04, *P* = 0.027) were significantly associated with increased major postoperative complications. The high/moderate risk GNRI group was also a significant predictor for prolonged PLOS (OR 3.91, *P* = 0.002).

**Conclusions:**

Preoperative GNRI has the potential to be a predictive tool for identifying high-risk elderly patients and monitoring nutritional status preoperatively to improve postoperative surgical outcomes following PD.

## Introduction

The relationship between malnutrition and increased postoperative complications has been well-established in the literature [1]. As life expectancy increases, the impact of malnutrition on elderly patients undergoing major surgeries has become a critical concern. The prevalence of malnutrition among hospitalized older adults was reported to be up to 50%, often associated with unfavorable surgical outcomes such as wound dehiscence, postoperative infections, longer hospital stays, and even increased mortality [2, 3].

Pancreatoduodenectomy (PD) is a potentially curative surgical option commonly employed in treating periampullary neoplasms. The safety of PD for elderly patients is a topic of debate due to the higher mortality and morbidity rates observed in this population [4–6].

Previous studies have found that factors such as comorbidities, advanced age, tumor staging, and nutritional status can significantly affect the outcomes of PD [7–9]. However, data regarding the association between preoperative malnutrition and postoperative outcomes in elderly patients undergoing PD is limited. The critical challenge might be the lack of an appropriate and reliable assessment tool of the nutritional status of elderly patients undergoing PD.

There are several methods to evaluate the nutritional status of elderly patients, including subjective global assessment (SGA), the Nutritional Risk Screening 2002 (NRS 2002), and the Mini Nutritional Assessment (MNA). However, these methods have limitations, such as highly relying on the subjective interpretation of clinical history and physical examination, leading to potential bias and time-consuming to conduct, which may not be ideal in a busy surgical setting. The Geriatric Nutritional Risk Index (GNRI), first introduced by Bouillanne et al., is a simple and objective nutritional screening tool specifically designed for the geriatric population [10]. It is calculated based on serum albumin levels and the actual to ideal body weight ratio. Multiple studies have shown GNRI to be a strong predictor of both morbidity and mortality in various patient populations, especially those with cancer surgery and emergency surgery [11–13]. Nevertheless, there is limited research regarding the correlation between preoperative GNRI and postoperative results in older patients who have undergone PD.

Our study aimed to examine the relationship between preoperative malnutrition, as measured by GNRI, and postoperative mortality and morbidity in elderly patients who undergo PD. Additionally, we sought to determine whether malnutrition is a noteworthy risk factor for major surgical complications and extended hospital stay after surgery.

## Methods

### Patient selection criteria and stratification

**Fig. 1:**
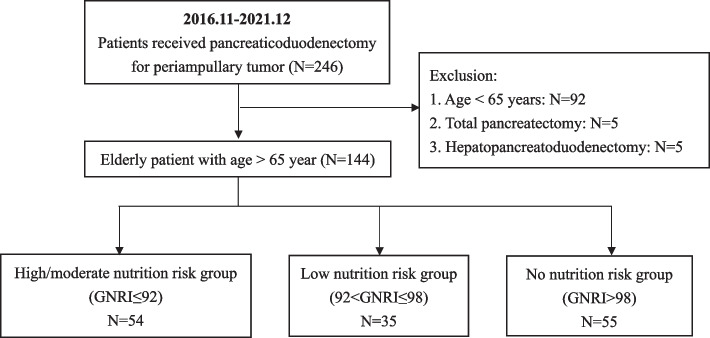
Flow chart of the patient selection process. *GNRI* Geriatric Nutritional Risk Index

This retrospective cohort study included adult patients who had undergone PD for periampullary tumors at Kaohsiung Chang Gung Memorial Hospital from November 2016 through December 2021. Periampullary tumors included periampullary benign lesions, ampulla vater adenocarcinoma, biliary adenocarcinoma, pancreatic adenocarcinoma, intraductal papillary mucinous neoplasm, neuroendocrine tumor, duodenal adenocarcinoma and other malignancy tumor. This study was approved by the Chang Gung Memorial Hospital Institutional Review Board. (Institutional Review Board approval no. 202301232B0). Patients under the age of 65 years who underwent total pancreatectomy and who underwent hepatopancreatoduodenectomy for oncologic reasons were all excluded from the analysis. To evaluate the nutritional status of the elderly patients within our cohort, we employed the GNRI as a quantifiable measure. The GNRI was determined using the subsequent formula: GNRI = 1.489 × serum albumin level (g/L) + [41.7 x (actual body weight/ideal body weight (IBW))]. In accordance with the original paper by Bouillanne et al., for patients with an actual body weight/IBW ratio greater or equal to 1, the ratio was set to 1. Serum albumin levels were obtained upon the patient’s admission to the ward or intensive care unit (ICU). Based on their calculated GNRI values, patients were stratified into three nutritional risk categories: high/moderate nutrition risk (GNRI ≤ 92), low nutrition risk (92 < GNRI ≤ 98), and no nutrition risk (GNRI > 98). The study flow chart is shown in Fig. 1.

### Surgical procedures and perioperative management

Surgical procedures of traditional open PD and minimally invasive PD, including laparoscopic and robotic approaches, were described previously [5]. There was no absolute contraindication in the minimally invasive approach except for patients with borderline resectable pancreatic adenocarcinoma who receive neoadjuvant chemotherapy. In that case, an open approach would be recommended.

Perioperative management included the preoperative, intraoperative, and postoperative elements. During the preoperative period, patients were routinely counseled about surgery and anesthesia to reduce anxiety with multimedia information. Bowel preparation was done for every patient who received PD due to the possibility of colon resection intraoperatively. Patients were encouraged to do physiotherapy and exercise training with Intermittent positive pressure and incentive spirometry. Preoperative biliary drainage will be arranged if total bilirubin is above 10 mg/dl or biliary tract infection or the patient will receive neoadjuvant chemotherapy. We do not use chemical thromboprophylaxis but mechanical measures, such as compression stockings or intermittent pneumatic compression, to prevent venous thromboembolism. Antimicrobial prophylaxis, skin preparation, and hypothermia avoidance were routinely performed intraoperatively. All patients received nasogastric (NG) tubes, foley catheters, and open drains to detect postoperative bleeding and leakage. In the postoperative period, patients remove the NG tube and sip water after flatus; it’s usually at postoperative day 3 (POD3). The diet formula will progress gradually if the patient has an appetite. Artificial nutrition support with total or peripheral parenteral nutrition was routinely prescribed for at least one week postoperatively. The usage will be prolonged if DGE, ileus, or anastomosis leak, which may delay enteral feeding, developed or in patients whose oral intake is less than 60% of the energy requirements after 1 week. Somatostatin analogs were applied postoperatively for 3 days and may extend to 7 days according to pancreas enzyme levels in drainage fluids. Early mobilization and rehabilitation were encouraged from POD1, and the urinary catheter will be removed if ambulant well.

### Surgical outcomes and postoperative complications

The primary endpoint of this study was in-hospital mortality and morbidity. Other surgical outcome measures were included in this study, such as operation time, intra-operative estimated blood loss, conversion rate, reoperation rate, unplanned ICU readmission and postoperative length of stay (PLOS). Documented postoperative complications encompassed postoperative pancreatic fistula (POPF), hemorrhage, delayed gastric emptying (DGE), intra-abdominal infection, and pulmonary complications. Fistula Risk Score (FRS) is also calculated to determine the risk of clinically relevant POPF following PD [14]. The clinically relevant POPF was categorized based on the definition outlined by the International Study Group on Pancreatic Fistula's 2016 edition [15] and only grade B and grade C POPF were recorded. The Intra-abdominal infection was defined as an organ/space surgical site infection provided by The United States Centers for Disease Control and Prevention (CDC) [16]. The severity of postoperative complications was classified using the Clavien-Dindo system. Mortality was defined as any death occurring during the postoperative admission period.

To further evaluate the predictors of short-term surgical outcomes, we particularly focused on major postoperative complications (defined as Clavien-Dindo grade ≥ IIIB) and prolonged PLOS (defined as greater than 28 days). We performed a univariate and multivariate analysis on these two outcomes.

### Statistical analyses

Statistical analyses were conducted using IBM SPSS Statistics for Windows, version 23.0 (IBM Corp., Armonk, NY, USA) and NCSS 10 software (NCSS Statistical Software, Kaysville, UT, USA). Categorical data were compared using the X^2^-test and Fisher’s exact test. We verified the normality of distribution for continuous variables with the Kolmogorov–Smirnov test. For normally distributed data, we expressed continuous variables as means with standard deviation, while for non-normally distributed data, we used the median with the interquartile range. We utilized ANOVA analyses and Kruskal- Wallis tests to analyze normally and non-normally distributed continuous variables between groups. We conducted univariate logistic regression analyses to examine the risk factors for major complications and prolonged postoperative length of stay. Variables that were significantly associated in the univariate analyses were then entered into the multivariate logistic regression to control for potential confounding. All tests were two-sided, and a P-value less than 0.05 was considered statistically significant.

## Results

We retrospectively reviewed 246 adult patients who had undergone PD for periampullary tumors at Kaohsiung Chang Gung Memorial Hospital from November 2016 through December 2021. After the exclusion of patients under 65 years (*N* = 92), who received total pancreatectomy (*N* = 5) and hepatopancreatoduodenectomy (*N* = 5), 144 elderly patients were eligible for inclusion. The demographic and patient characteristics are summarized in Table 1. In our cohort, we observed significant differences across different GNRI categories. Older age (*P* = 0.015), lower body mass index (BMI) (*P* = 0.012), inferior American Society of Anesthesiologists (ASA) physical status (*P* = 0.013), inferior Eastern Cooperative Oncology Group (ECOG) performance status (*P* = 0.016), lower albumin level (*P* < 0.001), higher total bilirubin (TBIL) level and more preoperative bile drainage (*P* < 0.001) were found in the high/moderate risk group patients. Conversely, sex, history of abdominal surgery, prevalence of hypertension, diabetes mellitus, chronic kidney disease, coronary artery disease, chronic obstructive pulmonary disease, and the Charlson Comorbidity Index (CCI) showed no significant variation across GNRI categories.

**Table 1:** *Baseline characteristics of patients undergoing pancreatoduodenectomy according to GNRI categories. (n* = *144)*

Characteristic		Total (*N* = 144)	High/moderate risk GNRI ≤ 92 (*N* = 54)	Low risk 92 < GNRI ≤ 98 (*N* = 35)	No risk GNRI > 98 (*N* = 55)	*P*-value
Age (years), median (IQR)		71 (9)	74.5 (10)^a^	71 (10)	70 (5)	**0.015**
Male, *n* (%)		84 (58.3%)	31 (57.4%)	18 (51.4%)	35 (63.6%)	0.509
BMI (kg/m^2^), mean ± SD		22.85 ± 3.45	22.11 ± 4.31^a^	22.68 ± 3.11	23.67 ± 2.44	**0.012**
Abdominal surgery history, *n* (%)		29 (20.1%)	13 (24.1%)	6 (17.1%)	10 (18.2%)	0.655
Hypertension, *n* (%)		74 (51.4%)	27 (50%)	19 (54.3%)	28 (50.9%)	0.921
Diabetes mellitus, *n* (%)		43 (29.9%)	14 (25.9%)	10 (28.6%)	19 (34.5%)	0.606
Chronic kidney disease, *n* (%)		12 (8.3%)	6 (11.1%)	2 (5.7%)	4 (7.3%)	0.625
Coronary artery disease, *n* (%)		27 (18.8%)	9 (16.7%)	7 (20%)	11 (20%)	0.884
Chronic obstructive pulmonary disease, *n* (%)		7 (4.9%)	4 (7.4%)	1 (2.9%)	2 (3.6%)	0.538
Disease						
Benign, n (%)		8 (5.6%)	2 (3.7%)	1 (2.9%)	5 (9.1%)	0.460^†^
Ampulla of vater adenocarcinoma, n (%)		41 (28.5%)	17 (31.5%)	10 (28.6%)	14 (25.5%)	0.784
Biliary adenocarcinoma, n (%)		22 (15.3%)	6 (11.1%)	8 (22.9%)	8 (14.5%)	0.317
Pancreatic adenocarcinoma, n (%)		55 (38.3%)	23 (42.6%)	12 (34.3%)	20 (36.4%)	0.688
Intraductal papillary mucinous neoplasm, n (%)		6 (4.2%)	3 (5.6%)	0 (0%)	3 (5.5%)	0.504^†^
Neuroendocrine tumor, n (%)		4 (2.8%)	0 (0%)	1 (2.9%)	3 (5.5%)	0.288^†^
Other malignancy, n (%)		8 (5.6%)	3 (5.6%)	3 (8.6%)	2 (3.6%)	0.524^†^
CCI, *n* (%)	0	66 (45.8%)	22 (40.7%)	19 (54.3%)	25 (45.5%)	0.114^†^
	1	47 (32.6%)	20 (37%)	7 (20%)	20 (36.4%)	
	2	24 (16.7%)	10 (18.5%)	8 (22.9%)	6 (10.9%)	
	3	4 (2.8%)	0 (0%)	0 (0%)	4 (7.3%)	
	4	2 (1.4%)	1 (1.9%)	1 (2.9%)	0 (0%)	
	5	1 (0.7%)	1 (1.9%)	0 (0%)	0 (0%)	
ASA physical status, *n* (%)	II	45 (31.3%)	11 (20.4%)^a^	9 (25.7%)	25 (45.5%)	**0.013**^**†**^
	III	98 (68.1%)	43 (79.6%)^a^	26 (74.3%)	29 (52.7%)	
	IV	1 (0.7%)	0 (0%)	0 (0%)	1 (1.8%)	
ECOG, *n* (%)	0	125 (86.8%)	41 (75.9%)^a^	31 (88.6%)	53 (96.4%)	**0.016**^**†**^
	1	16 (11.1%)	11 (20.4%)^a^	3 (8.6%)	2 (3.6%)	
	2	3 (2.1%)	2 (3.7%)	1 (2.9%)	0 (0%)	
GNRI score, mean ± SD		94.43 ± 9.75	84.30 ± 5.79^ab^	95.27 ± 1.61^a^	103.86 ± 4.85	** < 0.001**
Preoperative albumin (g/dL), mean ± SD		3.67 ± 0.63	3.05 ± 0.37^ab^	3.79 ± 0.48^a^	4.20 ± 0.32	** < 0.001**
Preoperative TBIL (mg/dL), median (IQR)		1.5 (2.7)	2.9(3.6)^a^	1.6(2.4)^a^	0.8(1.4)	** < 0.001**
Preoperative bile drain, *n* (%)		87 (60.4%)	38 (70.4%)^a^	28 (80%)^a^	21 (38.2%)	** < 0.001**

^†^*P*-values according to Fisher’s exact tests

^a^Significantly different versus No risk group by post-hoc comparison

^b^Significantly different versus Low risk group by post-hoc comparison

*GNRI* Geriatric Nutritional Risk Index, *IQR* interquartile range, *BMI* body mass index, *SD* Standard deviation, *CCI* Charlson Comorbidity Index, *ASA* American Society of Anesthesiologists, *ECOG* Eastern Cooperative Oncology Group, *TBIL* Total bilirrbun

Short-term surgical outcomes of patients undergoing PD were also compared according to nutrition risk defined by GNRI (Table 2). Patients in the high/moderate risk group had a statistically significantly higher mortality rate (11.1%, *P* = 0.024) compared to patients in the no-risk group, who experienced no mortality. A significantly longer median length of postoperative stays (*P* < 0.001), prolonged ICU stays (*P* = 0.001), prolonged TPN use (*P* = 0.001), delay days to ambulation (*P* = 0.006), and delayed drainage tube removal (*P* < 0.001) were also observed in the high/moderate risk group. Notably, the high/moderate risk group had a significantly higher incidence of over grade IIIB complications (37.0%, *P* = 0.001), unplanned ICU readmission (35.2%, *P* = 0.004) and pulmonary complications (20.4%, *P* = 0.009) than the other two groups. 18 (33.3%) patients in the high/moderate risk group experienced unplanned ICU readmission: six patients suffered from septic shock (5 pulmonary complications and 2 IAI); six patients suffered from internal bleeding; four patients suffered from anastomosis leakage; one patient due to bowel obstruction and one patient due to wound dehiscence. 4(11.4%) patients in the low nutrition risk group suffered from ICU readmission due to pulmonary complications (1 patient), internal bleeding (2 patients), and IAI with septic shock (1 patient). In the no nutrition risk group, patients were readmitted to ICU due to five internal bleeding, one anastomosis leak, and one catheter-related bloodstream infection with septic shock.

**Table 2:** *Short-term surgical outcomes of patients undergoing pancreatoduodenectomy according to GNRI categories (n* = *144)*

Characteristic		Total (*N* = 144)	High/moderate risk GNRI ≤ 92 (*N* = 54)	Low risk 92 < GNRI ≤ 98 (*N* = 35)	No risk GNRI > 98 (*N* = 55)	*P*-value
Operative time (minutes), median (IQR)		474.5 (210)	500 (223.75)	450 (182)	475 (230)	0.608
Operative blood loss (ml), median (IQR)		250 (300)	300 (325)	200 (250)	250 (250)	0.371
MIS approach, *n* (%)		28 (19.4%)	12 (22.2%)	6 (17.1%)	10 (18.2%)	0.832
Malignancy, *n* (%)		133 (92.4%)	50 (92.6%)	34 (97.1%)	49 (89.1%)	0.443
Tumor size (cm), median (IQR)		3 (2.15)	2.9 (2.275)	3 (1.325)	3.2 (2)	0.309
Mortality, *n* (%)		9 (6.3%)	6 (11.1%)^a^	3 (8.6%)	0 (0%)	**0.024**^**†**^
Readmission, *n* (%)		6 (4.2%)	3 (5.6%)	1 (2.9%)	2 (3.6%)	0.882^†^
Unplanned ICU Readmission, *n* (%)		29 (20.1%)	18 (33.3%) ^a^	4 (11.4%)	7(12.7%)	**0.009**
Reoperation,* n* (%)		27 (18.8%)	14 (25.9%)	6 (17.1%)	7 (12.7%)	0.202
Post-operative stays (days), median (IQR)		25 (14.75)	29.5 (19.25)^a^	25 (11)	22 (11)	** < 0.001**
Length of ICU stays (days), median (IQR)		4 (3)	5 (6)^a^	4 (3)	3 (3)	**0.001**
Soft diet (days), median (IQR)		8 (4)	8 (4)	8 (4)	7 (3)	0.097
Ambulation (days), median (IQR)		6 (3)	7 (6.5)^a^	6 (2)	5 (2)	**0.006**
TPN use (days), median (IQR)		9 (8)	12 (8.5)^a^	10 (9)^a^	7 (5)	**0.001**
Drain remove (days), median (IQR)		21 (11)	23 (17)^a^	21 (13)^a^	17 (9)	** < 0.001**
> Grade IIIb Complication, *n* (%)		30 (20.8%)	20 (37.0%)^ab^	5 (14.3%)	5 (9.1%)	**0.001**
Fistula Risk Score, median (IQR)		4 (4)	4 (3)	4 (3)	4 (3)	0.488
Pancreatic fistula, *n* (%)	B	14 (9.7%)	2 (3.7%)	3 (8.6%)	9 (16.4%)	0.082
	C	9 (6.3%)	4 (7.4%)	3 (8.6%)	2 (3.6%)	0.627^†^
Pulmonary complication, *n* (%)		16 (11.1%)	11 (20.4%)^a^	4 (11.4%)	1 (1.8%)	**0.009**
Intra-abdominal infection, *n* (%)		47 (32.6%)	18 (33.3%)	16 (45.7%)	13 (23.6%)	0.097
Postoperative bleeding, *n* (%)		15 (10.4%)	6 (11.1%)	4 (11.4%)	5 (9.1%)	0.944
Delay gastric emptying, *n* (%)		32 (22.2%)	14 (25.9%)	7 (20%)	11 (20%)	0.764

^†^*P*-values according to Fisher’s exact tests

^a^Significantly different versus No risk group by post-hoc comparison

^b^Significantly different versus Low risk group by post-hoc comparison

*GNRI* Geriatric Nutritional Risk Index *IQR* Interquartile range, *MIS* minimally invasive surgery, *ICU* Intensive care unit, *TPN* Total parenteral nutrition

There are no significant differences in operative time, operative blood loss, approach procedure, presence of malignancy, tumor size, readmission rate, reoperation rate, time to soft diet initiation, FRS, incidence of POPF, intra-abdominal infection, postoperative bleeding, and DGE.

14 (25.9%) patients in the high/moderate risk group experienced reoperation: seven patients for check bleeding by laparotomy or angiography; four patients received anastomosis revision due to leakage; one received tracheostomy for prolonged ventilator support, and two patients received laparotomy due to bowel obstruction and wound dehiscence, respectively. In the low nutrition risk group, one received tracheostomy, four patients reoperated for check bleeding by laparotomy or angiography, and one patient received surgical intervention for intra-abdominal abscess formation. In the no nutrition risk group, five patients reoperated for internal bleeding, one received anastomosis revision, and one patient received laparotomy for IAI.

The results of both univariate and multivariate analyses investigating predictors of major postoperative complications (Clavien-Dindo grade ≥ IIIB) are shown in Table 3. In the univariate analysis, the high/moderate risk GNRI group exhibited a significantly higher risk for major postoperative complications (OR 5.88, 95% CI 2.01–17.19, *P* = 0.001). Advanced age (OR 1.14, 95% CI 1.06–1.23, *P* < 0.001), an ASA score greater than 3 (OR 8.48, 95% CI 1.92–37.39, *P* = 0.004), and operative time exceeding 8 h (OR 2.99, 95% CI 1.26–7.09, *P* = 0.013) were also identified as significant predictors for major complications. In multivariate analysis, the high/moderate risk GNRI group remained significantly associated with increased risk for major postoperative complications (adjusted OR 3.61, 95% CI 1.12–11.69, *P* = 0.032). Increased age (adjusted OR 1.11, 95% CI 1.02–1.21, *P* = 0.014) and operative time over 8 h (adjusted OR 3.04, 95% CI 1.14–8.13, *P* = 0.027) were also confirmed to be associated with increased complications.

**Table 3:** *Univariate and multivariate analysis of predictors for major postoperative complications(Clavien-Dindo grade* ≥ *IIIb)*

Variables	Unadjusted OR (95% CI)	*P*-value	Adjusted OR (95% CI)	*P*-value
**Age**	**1.14 (1.06–1.23)**	** < 0.001**	**1.11 (1.02–1.21)**	**0.014**
Sex (Female)	0.62 (0.28–1.49)	0.300	—	
BMI	0.98 (0.87–1.10)	0.758	—	
Preoperative TBIL	1.03 (0.93–1.14)	0.546		
Preoperative bile drain	0.59 (0.25–1.40)	0.231		
CCI				
0–1	Reference		—	
2–5	0.89 (0.33–2.42)	0.819	—	
**ASA physical status**				
**I-II**	**Reference**		**Reference**	
**III-V**	**8.48 (1.92–37.39)**	**0.004**	4.56 (0.95–21.94)	0.058
**GNRI score**			**—**	
** No risk**	**Reference**		**Reference**	
Low risk	1.67 (0.45–6.24)	0.448	1.35 (0.33–5.57)	0.678
** High/moderate risk**	**5.88 (2.01–17.19)**	**0.001**	**3.61 (1.12–11.69)**	**0.032**
MIS approach^a^	0.79 (0.27–2.29)	0.666	—	
** Operative time**				
Time < 8 h	Reference		Reference	
**Time > 8 h**	**2.99 (1.26–7.09)**	**0.013**	**3.04 (1.14–8.13)**	**0.027**
Operative blood loss				
< 400 ml	Reference		—	
> 400 ml	1.95 (0.82–4.63)	0.126	—	
Fistula Risk Score	1.14 (0.94–1.39)	0.192	—	

^a^Compared to open approach

*OR* odds ratio, *CI* confidence interval, *TBIL* Total bilirubin, *BMI* body mass index, *CCI* Charlson Comorbidity Index, *ASA* American Society of Anesthesiologists, *GNRI* Geriatric Nutritional Risk Index, *MIS* minimally invasive surgery

High risk / moderate nutrition risk (GNRI ≤ 92), low nutrition risk (92 < GNRI ≤ 98), and no nutrition risk (GNRI > 98)

The results of univariate and multivariate analyses of predictors for PLOS were summarized in Table 4 and revealed malnutrition and advanced age may be associated with prolonged hospital stays. In the univariate analysis, the high/moderate risk group was significantly associated with prolonged PLOS (OR 4.64, 95% CI 1.98–10.86, *P* < 0.001). Besides, advanced age is also significantly associated with PLOS (OR 1.09, 95% CI 1.02–1.16, *P* = 0.010). Other factors, including sex, BMI, CCI, ASA score, operative time, operative blood loss and RFS, did not reach statistical significance. In the multivariate analysis, the high/moderate risk GNRI group retained the only significant predictor for prolonged PLOS (adjusted OR 3.91, 95% CI 1.63–9.37, *P* = 0.002).

**Table 4:** *Univariate and multivariate analysis of predictors for prolonged postoperative hospital stays (*> *28 days)*

Variables	Unadjusted OR (95% CI)	*P*-value	Adjusted OR (95% CI)	*P*-value
**Age**	**1.09 (1.02–1.16)**	**0.010**	1.06 (0.99–1.13)	0.086
Sex (Female)	0.81 (0.41–1.63)	0.558	—	
BMI	1.03 (0.93–1.14)	0.551	—	
Preoperative TBIL	1.03 (0.94–1.13)	0.499		
Preoperative bile drain	0.72 (0.36–1.46)	0.360		
CCI				
20–1	Reference		—	
2–5	1.63 (0.72–3.65)	0.239	—	
**ASA physical status**				
**I-II**	Reference		—	
**III-V**	1.86 (0.86–4.04)	0.114	—	
**GNRI score**				
No risk	Reference		Reference	
Low risk	2.09 (0.80–5.46)	0.134	1.94 (0.73–5.14)	0.180
**High/moderate risk**	**4.64 (1.98–10.86)**	** < 0.001**	**3.91 (1.63–9.37)**	**0.002**
MIS approach^a^	0.42 (0.16–1.10)	0.077	—	
Operative time				
Time < 8 h	Reference		—	
Time > 8 h	1.33 (0.67–2.63)	0.413	—	
Operative blood loss				
< 400 ml	Reference		—	
> 400 ml	2.03 (0.94–4.35)	0.068	—	
Fistula Risk Score	1.14 (0.96–1.34)	0.131	—	

^a^Compared to open approach

*OR* odds ratio, *CI* confidence interval, *TBIL* Total bilirubin, *BMI* body mass index *CCI* Charlson Comorbidity Index, *ASA* American Society of Anesthesiologists, *GNRI* Geriatric Nutritional Risk Index, MIS minimally invasive surgery

High risk / moderate nutrition risk (GNRI ≤ 92), low nutrition risk (92 < GNRI ≤ 98), and no nutrition risk (GNRI > 98)

## Discussion

In this study, we analyzed the association between preoperative GNRI and postoperative outcomes in elderly patients undergoing PD. Our study cohort comprised 144 patients over 65 years old, and we observed a strong and significant association between lower preoperative GNRI values and increased mortality rates, a higher incidence of major complications, and delayed postoperative recovery. Furthermore, the multivariate analysis further confirmed the independent association of lower GNRI score with major postoperative complications (Clavien-Dindo grade ≥ IIIB) and prolonged hospital stays (> 28 days). These results have significant implications for clinical practice, as they emphasize the necessity of identifying and mitigating malnutrition risk factors preoperatively, which may improve surgical outcomes.

The elderly population is at a potential risk for malnutrition, frequently linked to physical, psychological, or physiological dysfunction associated with the aging process [17]. Additionally, the impact of malnutrition extends beyond its physiological consequences. It contributes to an increased risk of decline in quality of life, performance status, and resistance to infections due to inferior immune function [18]. Elderly patients suffering from gastrointestinal cancer are particularly susceptible to malnutrition [19, 20] due to the mechanical hinderance of oral food intake and the impairment of digestion caused by local or systemic impacts of the disease. Furthermore, malnutrition has been widely reported as a significant predictor for decreased survival of gastrointestinal cancer [21, 22].

Studies have shown that preoperative nutrition status can influence the occurrence of complications following PD and other gastrointestinal surgery [23–25]. The study from Eunjung et al. examined clinical outcomes based on the Mini Nutrition Assessment in patients who underwent PD [24]. The patients who were malnourished or at risk of malnutrition experienced more complications and pancreatic fistulas than their well-nourished counterparts. Masaki et al. found that among a set of preoperative nutritional variables, a cholinesterase concentration of less than 250 IU/L was the only independent predictive factor for the incidence of higher postoperative complications in patients who underwent PD [23]. However, the studies focusing on preoperative malnutrition status in elderly patients undergoing PD, especially using GNRI as a preoperative evaluation tool, are still limited. Several studies revealed lower GNRI might associated with surgical site infection or postpancreatectomy hemorrhage after PD [26, 27]. Our study contributes to the existing literature by highlighting the robustness and clinical relevance of preoperative GNRI assessment in predicting adverse outcomes after PD in elderly patients. In our multivariate analysis, patients in the high/moderate risk group might suffer from relatively high in-hospital mortality (11.1%), prolonged PLOS, and major complications.

Recently, minimally invasive surgery (MIS) has been adopted for treating pancreatic malignancies due to its safety and feasibility, even in elderly patients [5]. Paiella, Salvatore et al. reported that there was no difference between MIS versus open approach in complications of patients older than 70 years undergoing distal pancreatectomy [28]. Interestingly, they also identified fragile elderly stratified according to the modified frailty index are more prone to develop postoperative complications. In our cohort, there was about 20% of patients received MIS. The MIS approach seems to have better outcomes, including fewer major complications and hospital stays. However, there was no statistical difference during the analysis, and further randomized controlled studies may be needed.

Multiple nutrition screening tools are available to assess perioperative nutritional status, including BMI, prognostic nutritional index (PNI), SGA, and skeletal muscle index. Nevertheless, applying these tools to elderly patients undergoing PD is constrained due to the limitations of those tools and the absence of a consensus regarding their effectiveness. For example, based on the serum albumin and total lymphocyte count, PNI might be a valuable measure of nutritional and immune status but less sensitive to changes in the nutritional status of geriatric patients [29]. SGA assesses the nutritional status based on medical history and physical examination, requiring expert knowledge and difficulty presenting the real-time nutrition status [30]. The measurement of skeletal muscle index requires imaging techniques such as dual-energy X-ray absorptiometry (DXA), bioelectric impedance vector analysis (BIVA), computed tomography, or magnetic resonance imaging scans, which may not be routinely available and could be an extra burden for patients [31]. However, recent studies have revealed that preoperative sarcopenia and sarcopenic obesity may affect the outcomes in patients who received PD for pancreatic cancer, especially in elderly [32, 33]. It is mandatory that body composition evaluation should be integrated as a comprehensive assessment for preoperative nutrition risk stratification.

On the other hand, the GNRI offers distinct advantages as an objective and readily accessible predictive tool that specifically addresses the nutritional risk of morbidity and mortality in elderly patients. Its classification value has already been recognized, and the measured parameters, such as serum albumin, body height, and body weight, have been routinely assessed in cancer patients [10]. Previous studies have identified GNRI as a prognostic predictor for hospital length of stays and chronic diseases among elderly patients with sepsis, acute respiratory distress syndrome, or traumatic fall [34–36]. More recently, GNRI has emerged as a valuable predictor for morbidity and mortality in patients with cancer. Masaru et al. reported that the patients in the all-risk group (GNRI ≤ 98) are associated with increased postoperative complications and poor prognosis in elderly patients with colorectal cancer after curative surgery than patients with the no-risk group (GNRI > 98) [37]. Noriyuki et al. found that the GNRI is significantly associated with overall survival (OS) and cancer-specific survival (CSS) in elderly gastric cancer patients who underwent laparoscopic gastrectomy with R0 resection [11]. Naotake et al. reported that a GNRI value below 99 was significantly associated with an increased risk of postoperative complications after curative pancreatic resection [38]. Furthermore, both univariate and multivariate analyses demonstrated a significant association between a GNRI value below 99 and longer OS [38]. The findings of these studies support our result that high/moderate nutrition risk (GNRI ≤ 92) is a significant factor associated with a higher incidence of major complications and prolonged postoperative hospital stays in elderly patients undergoing PD.

POPF is one of the most severe complications after PD, caused by pancreatic juice leakage into the abdomen, which sometimes leads to intraabdominal abscess formation and subsequent internal lethal hemorrhage. The overall incidence rate of CR-POPF in our cohort is 16.0%, which is similar to the previous systemic review [39].

However, our study showed a relatively higher rate of POPF in the No Risk group, which is not coherent with the current literature but is not statistically significant. Naotake et al. mentioned that GNRI of < 94 was significantly associated with surgical site infections (*P* < 0.001). which was occasionally due to POPF [26] and GNRI of < 92 was an independent risk factor of postpancreatectomy hemorrhage after PD [27]. Therefore, We used FRS to evaluate this patient further and disclosed that there was no significant difference between these groups. Although the risk factor of POPF is multifactorial and difficult to predict, FRS is currently the most validated and accurate prediction tool for CR-POPF. Accordingly, it’s reasonable that there is no difference in POPF. We assume FRS has more impact on POPF than nutrition risk. Besides, GNRI was calculated with only albumin level and the actual to ideal body weight ratio and doesn’t include the body component analysis, which may affect the outcome of these patients. In addition, the reason for discordance may be the small sample size and the timing of stratified these patients by the albumin level obtained upon admission without continuous monitoring. Further investigation into predicting POPF is needed.

There are several limitations of our study. firstly, it was a retrospective, single-center study with a relatively small data size that may result in bias in data analysis and possibly overlook the effect of GNRI. Secondly, the study does not compare results using other common nutritional assessment tools such as PNI, SGA, MNA, skeletal muscle index, and the Malnutrition Universal Screening Tool. Whether GNRI remains the superior predictive tool among nutritional assessment indicators cannot be determined from our study alone. Our study primarily focuses on short-term surgical outcomes without providing much information on the long-term effects of preoperative nutrition status on patient recovery and survival. To strengthen the evidence and validate the significance of GNRI in elderly patients undergoing PD, it is essential to conduct further prospective and larger scale studies to assess the role of GNRI and its impact on clinical outcomes of patients undergoing PD.

## Conclusions

In conclusion, our study provides robust evidence associating preoperative malnutrition and postoperative short-term results after PD. A lower GNRI (≤ 92), reflective of high and moderate nutritional risk, is independently linked to increased incidences of major complications and longer PLOS in patients over 65 years old. Preoperative GNRI serves as a potential tool for identifying elderly patients at high risk for morbidity and mortality after PD. The value of GNRI predicting outcomes expected to grow in the future.

## Data Availability

The data that support the findings of this study are not openly available due to reasons of sensitivity and are available from the corresponding author upon reasonable request. Data are located in controlled access data storage at KCGMH.

## Abbreviations

ASA: American Society of Anesthesiologists
BMI: Body mass index
CCI: Charlson Comorbidity Index
DGE: Delayed gastric emptying
ECOG: Eastern Cooperative Oncology Group
FRS: Fistula Risk Score
GNRI: Geriatric Nutritional Risk Index
IBW: Ideal body weight
PD: Pancreatoduodenectomy
PLOS: Postoperative length of stay
POPF: Postoperative pancreatic fistula
SGA: Subjective global assessment
TBIL: Total bilirubin
